# Identifying the fresh vegetables foodshed of Brazzaville: A new dataset from a market-based survey

**DOI:** 10.1016/j.dib.2026.112989

**Published:** 2026-06-20

**Authors:** Joaquin Ameller, Prince Loïque Maba Ngouloubi, Paule Moustier

**Affiliations:** a CIRAD, UMR MoISA, F-34398 Montpellier, France; b MoISA, Univ Montpellier, CIHEAM-IAMM, CIRAD, INRAE, Institut Agro, IRD, Montpellier, France; c Institut Supérieur des Sciences Géographiques, Environnementales et de l’Aménagement (ISSGEA), Université DENIS SASSOU-N’GUESSO, Brazzaville, Republic of the Congo

**Keywords:** Food flows, Foodshed, Vegetable supply chain, Market survey

## Abstract

This dataset documents vegetable supply flows in Brazzaville, compiled through a targeted survey of wholesale and retail vendors operating in the city’s main markets. The survey aimed to capture information on 16 vegetables inflows during one day in dry and rainy seasons. Data describes food flows in terms of value, volume, and origins including production sites, regions and intermediary vendors if applicable.

The dataset was produced using field-based questionnaires. Surveyors conducted in-person interviews with market vendors, recording prices, quantities and details on sourcing locations. The resulting data provides harmonized variables describing food flows and supply nodes.

This new dataset offers a detailed mapping of the channels through which vegetables reach the city of Brazzaville and provides a basis for examining spatial patterns of supply and vendor networks. Such data had not been produced since the 1990s. The dataset can be reused for research and planning analyses related to food provisioning, food system organization, transport logistics, and market dynamics. It also offers potential for comparative work with similar datasets from other urban contexts, especially in Central African cities, as well as for integration into modelling exercises exploring food flows and market accessibility.

Specifications Table

**Table:** 

Subject	Social Sciences
Specific subject area	Agriculture & Food economics.
Type of data	CSV data file. Raw, Analysed.
Data collection	Data were collected through field surveys across the major wholesale and retail markets of Brazzaville, following a sampling plan covering 216 wholesalers and 1109 retail vendors. The entire data collection was carried out on one single day per season, once during the rainy season and once during the dry season, to ensure full comparability of prices and product origins across all markets. The dataset was generated using structured questionnaires, direct observations, and product weighing.
Data source location	City: Brazzaville Country: Republic of the Congo Latitude and longitude: 0.8°S latitude and 14.3°E longitude.
Data accessibility	Repository name: Cirad Dataverse. Dataset of produce supply flows in Brazzaville: A survey of traders. Data identification number: 10.18167/DVN1/DAMXAW Direct URL to data: https://doi.org/10.18167/DVN1/DAMXAW
Related research article	Moustier, P., Ameller, J., Maba Ngouloubi, P. L. (Working Paper). Permanence and change in Brazzaville vegetable supply: the role of logistics. 1–12. Montpellier, Cirad.

## 1 Value of the Data

• Cities worldwide are increasingly engaged in characterizing their foodsheds to assess sustainability and resilience opportunities. This dataset provides information on vegetable flows collected through a survey of >1300 traders operating across retail and wholesale markets.
• In contexts such as Brazzaville, where trade commonly relies on non-standard units (e.g., buckets, bags, handfuls), these data offer a reliable record of food flow quantities expressed in standardized measures. They can support evidence-based decision-making for urban planning and food policy.
• These data can be reused for research purposes to examine spatial patterns of food sourcing, supply chain organization, urban market dynamics, structure of informal food systems, and nutritious food availability.

## 2 Background

In many African cities, limited knowledge of food flows results from the informal and highly seasonal nature of food trade, the frequent use of non-standard measurement units, and the diversity of supply zones covering urban, rural, and international origins. This context requires methodological adaptations and a detailed focus on specific food groups. Improved empirical data on the origin, quantities, and circulation of food products are increasingly recognized as essential for analysing urban food systems and their resilience, food security implications, and sustainability [[1], [2], [3], [4], [5], [6]]. Datasets documenting food flows at the market level therefore provide inputs for food systems analysis and urban foodshed assessments.

To address this need, we conducted a survey directly at city markets in Brazzaville. The survey records the origins, prices, and volumes of vegetable products, covering all wholesalers and approximately one-quarter of vegetable retailers operating in the surveyed markets. This dataset contributes to ongoing efforts to map and understand urban foodsheds.

This dataset complements alternative approaches used to characterize food flows. For instance, estimates based on regional production and import statistics often underestimate the flows of perishable and highly seasonal products and provide limited information on how and where these volumes circulate within cities. Other survey approaches, such as roadside or checkpoint surveys, often capture intermediate nodes of food supply chains and provide limited information on final destinations. By documenting the origins, prices, and traded quantities of 16 vegetable products, this dataset provides empirical evidence that can be used to estimate and trace food flows supplying Brazzaville.

## 3 Data Description

This repository contains three files describing vegetable flows observed on urban markets in Brazzaville in 2022. The data document transactions observed on retail and wholesale markets during two survey periods corresponding to the rainy and dry seasons. The repository includes two datasets and one file describing the variables.

The files are:

• dt_produce_flows_retail_market.csv
• dt_produce_flows_wholesale_market. csv
• dictionnary_of_variables.xls

The dataset documents vegetable flows observed on urban markets through two complementary survey instruments. Observations were conducted during two seasons in 2022:

• rainy season (April 2022)
• dry season (August 2022)

The dataset covers 14 retail markets and 7 wholesale markets in Brazzaville. Some markets appear in both datasets when they host both retail and wholesale activities.

The vegetables included in the surveys are:

• Amaranth leafs (Amarante)
• African nightshade (Grande morelle)
• Bitter nightshade (Morelle amère)
• Hibiscus or Roselle (Oseille de Guinée)
• Hot pepper (Piment)
• Spring onion (Ciboule)
• Lettuce (Laitue)
• Cabbage (Chou pommé)
• Tomato (Tomate)
• Carrot (Carotte)
• Eggplant (Aubergine violette)
• Green bell pepper (Poivron vert)
• Zucchini (Courgette)
• Cucumber (Concombre)
• Malabar spinach (Baselle)
• Green bean (Haricot vert)

Observations were recorded at the level of product transactions observed on markets on the day of the survey. Observations are reported at the level of products sold on markets. The datasets do not contain any personal identifiers of vendors.

**Table:** Files description.

File	Variables

dt_produce_flows_retail_market.csv (82,028 observations).	• the season of observation • the market where the product was sold • the product type • the type of selling location on the market • the origin of the product • the sourcing place and supplier type • the purchase expenditure reported by the retailer • the transaction unit used for selling the product • calculated prices and volumes in kilograms
dt_produce_flows_wholesale_market.csv (102 observations)	• the market where the product was observed • the season of observation • the product type • the transaction unit used • the number of transaction units observed • the price per transaction unit • the weight of transaction units • the number of wholesalers present • the origin of the products (department of production)
dictionnary_of_variables.xls	• variable name • English variable label • description of the variable • data type • value labels where applicable

**Fig. 1:**
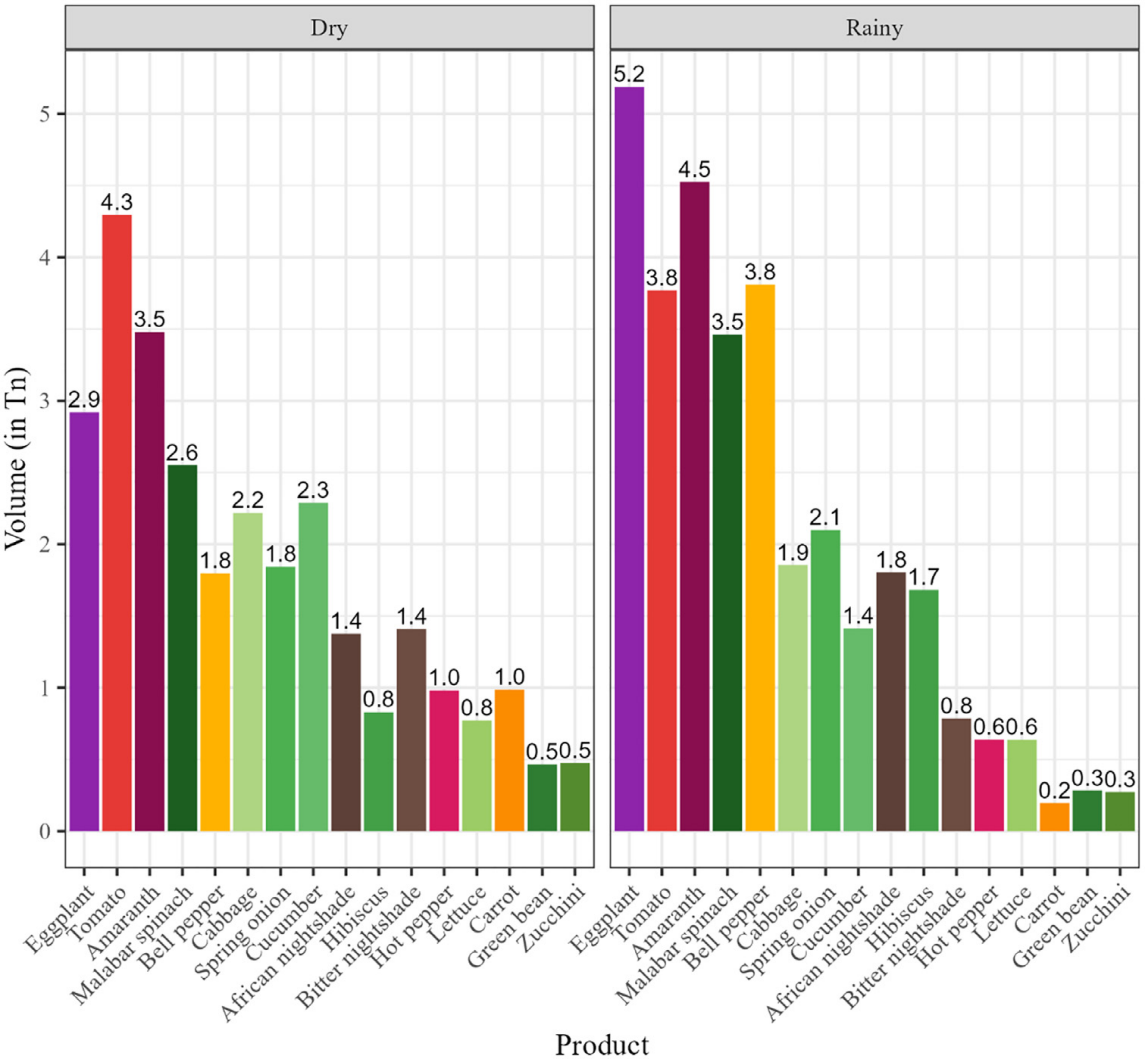
One-day aggregated flows (ton) circulating through retail markets per product per season.

The data enables calculating and tracing produce flows provisioning Brazzaville. Fig. 1 shows the aggregated volumes (ton) flowing through the retail markets as captured by our survey. Table 1 illustrates, for tomatoes and amaranth, how data allows to trace in what proportions the different regions of Congo contribute to those food flows.

**Table 1:** Origins by region of tomatoes and amaranth provisioning retail markets in Brazzaville.

Season	Product	Origin: Region	Region/Product (%)^I^	Product/Region (%)^II^
Dry	Amaranth	Brazzaville	87.02%	20.33%
Dry	Amaranth	Niari	0.01%	0.02%
Dry	Amaranth	Pool	12.96%	4.99%
Rainy	Amaranth	Bouenza	0.83%	9.27%
Rainy	Amaranth	Brazzaville	74.84%	19.06%
Rainy	Amaranth	Niari	0.29%	2.64%
Rainy	Amaranth	Plateaux	0.07%	0.21%
Rainy	Amaranth	Pool	23.47%	9.07%
Rainy	Amaranth	RDC^III^	0.50%	15.27%
Dry	Tomato	Bouenza	0.42%	6.04%
Dry	Tomato	Brazzaville	38.46%	10.19%
Dry	Tomato	Cuvette	0.83%	54.63%
Dry	Tomato	Niari	9.40%	17.45%
Dry	Tomato	Plateaux	1.37%	16.87%
Dry	Tomato	Pool	48.43%	21.14%
Dry	Tomato	RDC	1.10%	26.12%
Rainy	Tomato	Bouenza	0.40%	3.71%
Rainy	Tomato	Brazzaville	37.54%	7.96%
Rainy	Tomato	Cameroun	0.40%	100.00%
Rainy	Tomato	Niari	2.22%	16.59%
Rainy	Tomato	Plateaux	7.40%	18.49%
Rainy	Tomato	Pointe Noire	0.03%	0.59%
Rainy	Tomato	Pool	52.03%	16.75%

^I^ Contribution of a given region to the total volume of a given product flow. A few respondents did not disclose origins during the dry season, these observations were excluded for the calculations of this table. ^II^ Proportion of a given product to the total volume provided by a given region. ^III^ Democratic Republic of Congo.

## 4 Experimental Design, Materials and Methods

Data were collected through structured market surveys conducted in Brazzaville, Republic of the Congo, during one day in each of the two seasonal rounds (April and August 2022). The objective was to document the origin, quantities, and prices of selected vegetables traded in urban wholesale and retail markets.

An initial inventory effort enabled identifying 58 retail markets operating in Brazzaville. From this inventory, 14 markets were selected to capture the geographic distribution of markets across the city and variation in market size and customers’ profiles. The surveyed retail markets were: Total, Ouenzé, Texaco-La Tsiémé, Moungali, Poto-Poto, Talangaï, Mikalou, Mfilou, Bacongo, Makélékélé, Marché Plateau, Marché Commission, Marché Bourreau, and Marché Tsiémé. They represent the third of the total of retail market vendors. In addition, all the six wholesale markets supplying the city were surveyed: Commission, Bourreau, Hugos, Coaster, Texaco-Tsiémé, and Kibéliba.

In each market, traders selling one or more of the selected vegetables were approached and invited to participate in the survey. Data were collected using structured questionnaires administered to traders. The questionnaires recorded the product sold, its origin, the unit of sale, the number of units traded, and the observed price per unit on the day of the survey. Separate questionnaires were used for wholesale and retail markets to account for differences in trading practices.

Because vegetables are sold using heterogeneous units of transaction (e.g., baskets, crates, bundles), representative sales units were weighed during the survey to convert observed prices and declared expenses into standardized kilogram equivalents. For each product, several units of sale were weighed across vendors to estimate the average weight of the transaction unit used in the market. These measurements were then used to derive quantities and prices expressed per kilogram.

Completed questionnaires were coded and entered into a structured database using a standardized data-entry template. Data cleaning procedures included verification of product names, units of sale, and price consistency across observations before generating the final dataset.

## Limitations

The dataset has several limitations related to the scope and timing of the surveys. First, surveys were conducted on a single day during each of the two survey rounds. As a result, the dataset provides a snapshot of vegetable trade for a typical market day in each season. While this design allows estimation of market flows through extrapolation, it does not capture potential day-to-day or short-term temporal variations in quantities traded, prices, or supply origins within each season.

Second, the dataset is limited to the selected sample of wholesale and retail markets surveyed in Brazzaville. Although markets were chosen to reflect the geographic distribution and diversity of market types in the city, the dataset does not cover all markets operating in Brazzaville.

Third, the dataset focuses on vegetables traded through traditional wholesale and retail markets and does not capture vegetable flows circulating through supermarkets, restaurants, or other retail channels. Consequently, the dataset represents the structure and characteristics of vegetable supply chains operating through open markets rather than the entirety of the urban food distribution system.

## Ethics Statement

The authors confirm that this study complies with the ethical guidelines outlined in Data in Brief’s Guide for Authors.

For the voluntary participation of surveyed vendors, informed verbal consent was obtained prior to participation. Participants were informed about the non-commercial, academic purpose of the data collection. No personally identifiable information (PII) was recorded or stored.

## CRediT Author Statement

**Joaquin Ameller:** Validation, Formal analysis, Data Curation, Writing - Original Draft, Visualization. **Prince Loïque Maba Ngouloubi:** Methodology, Investigation, Writing - Review & Editing. **Paule Moustier:** Conceptualization, Methodology, Validation, Writing - Review & Editing, Supervision, Project administration, Funding acquisition.

## Data Availability

DataverseDataset of produce supply flows in Brazzaville: A survey of traders in 2022. (Original data)

## Appendix Supplementary materials

Image, application 1

Image, application 2
