# Supplementary material for: Identifying the fresh vegetables foodshed of Brazzaville: A new dataset from a market-based survey
Source: Data Brief. 2026 Jun 20;67:112989. doi: 10.1016/j.dib.2026.112989 (PMC13320381; doi:10.1016/j.dib.2026.112989)
Supplement: Supplementary file 1 [file mmc1.docx]

**Variables and labels translations**

**Marché gros / Wholesale market**

**1. season**

English name: Season

Description: Season of data collection

Data type: Categorical

Values and labels:

ss: Dry season

sp: Rainy season

**2. nom_marche**

English name: Market name

Description: Name of the market where data was collected

Data type: String

**3. type_produit**

English name: Product type

Description: Type of product surveyed

Data type: String

Values and labels:

Amarante: Amaranth.

Grande morelle: African nightshade.

Morelle amère: Bitter nightshade.

Oseille de Guinée: Hibiscus.

Piment: Hot pepper.

Ciboule: Spring onion.

Laitue: Lettuce.

Chou pomme: Cabbage.

Tomate: Tomato.

Carotte: Carrot.

Aubergine violette: Eggplant.

Poivron: Bell pepper.

Courgette: Zucchini.

Concombre: Cucumber.

Baselle: Malabar spinach.

Haricot vert: Green bean.

**4. unite_de_transaction_vente**

English name: Unit of sale

Description: Unit used for product transactions

Data type: Categorical

Values and labels:

Botte: Bundle

Seau: Bucket

Tas: Pile

Planche: bed of crops still in the field

**5. poids_kg**

English name: Weight (kg)

Description: Weight of the product

Data type: Numeric

Unit: Kilograms (kg)

**6. nb_unite**

English name: Number of units

Description: Number of units sold or observed

Data type: Numeric

**7. prix_ut**

English name: Unit price

Description: Price per unit

Data type: Numeric

Unit: FCFA per unit

**8. valeur_total_f_cfa**

English name: Total value (FCFA)

Description: Total transaction value

Data type: Numeric

Unit: FCFA

**9. volume_total_kg**

English name: Total volume (kg)

Description: Total quantity expressed in kilograms

Data type: Numeric

**10. nb_grossistes**

English name: Number of wholesalers

Description: Number of wholesalers involved

Data type: Numeric

**11. origine_produit**

English name: Product origin (1)

Description: Primary origin of the product

Data type: String

**Marché detail / Retail market**

**1. season**

English name: Season

Description: Agricultural season during which the observation was made

Data type: Categorical

Values and labels:

ss: Dry season

sp: Rainy season

**2. code_obs**

English name: Observation ID

Description: Unique identification code assigned to each observation

Data type: Numeric

**3. type_marche**

English name: Market type

Description: Type of market where the transaction takes place

Data type: Categorical

Values and labels:

Detail: Retail market

Gros: Wholesale market

**4. nom_marche**

English name: Market name

Description: Name of the market where the observation was recorded

Data type: String

**5. fonction_enquete**

English name: Respondent role

Description: Function or professional role of the respondent in the market

Data type: Categorical

Values and labels:

Détaillant: Retailer.

Producteur: Producer.

Demi-grossiste: Semi-wholesaler.

Grossiste: Wholesaler.

Grossiste / Détaillant: Wholesaler/Retailer.

Producteur / Demi-grossiste: Producer/Semi-wholesaler.

Détaillant / Producteur: Retailer/Producer.

Autoproducteur: Self-producer.

**6. sexe_enquete**

English name: Respondent gender

Data type: Categorical

Values and labels:

Masculin: Male

Feminin: Female

**7. type_loc**

English name: Selling location type

Description: Physical location or display mode where the product is sold within the market (e.g., table, ground)

Data type: Categorical

Values and labels:

Sur table: on table.

Par terre: On the floor.

Ambulant: Mobile.

**8. type_produit**

English name: Vegetable type

Description: Name of the vegetable being sold

Data type: String

Values and labels:

Amarante: Amaranth.

Grande morelle: African nightshade.

Morelle amère: Bitter nightshade.

Oseille de Guinée: Hibiscus.

Piment: Hot pepper.

Ciboule: Spring onion.

Laitue: Lettuce.

Chou pomme: Cabbage.

Tomate: Tomato.

Carotte: Carrot.

Aubergine violette: Eggplant.

Poivron: Bell pepper.

Courgette: Zucchini.

Concombre: Cucumber.

Baselle: Malabar spinach.

Haricot vert: Green bean.

**9. origine_produit_site**

English name: Production site

Description: Specific site of production of the product

Data type: string

**10. origine_produit_region**

English name: Production region

Description: Administrative or geographic region where the product was produced

Data type: string

**11. type_lieu_achat**

English name: Source location type

Description: Type of place where the product was sourced (e.g., farm, market, wholesaler)

Data type: Categorical

Values and labels:

Marche: Market.

Jardin: Garden.

Village: Village.

NA: Not available.

Autres: Others.

IAC: IAC.

Port: Port.

**12. nom_lieu_achat**

English name: Source location name

Description: Name of the place where the product was purchased by the respondent

Data type: String

**13. type_fournisseur**

English name: Supplier type

Description: Function or role of the supplier within the supply chain

Data type: Categorical

Values and labels:

Détaillant: Retailer.

Demi grossiste: Semi-wholesaler.

Producteur: Producer.

Grossiste: Wholesaler.

Auto producteur: Self-producer

**14. depense_achat_f_cfa**

English name: Purchase expenditure (CFA)

Description: Amount spent by the retailer to purchase the produce sold on the day of the survey

Data type: Numeric

Unit: FCFA

**15. unite_de_transaction**

English name: Sales transaction unit

Description: Unit in which the product is sold in the observed transaction

Data type: Categorical

Values and labels:

Botte: Bundle

Seau: Bucket

Tas: Pile

Planche: Bed of crops (in-field harvest unit)

Piece: Piece

Sac: Bag

Caisse: Crate/box

**16. prix_moyen_kg**

English name: Average selling price per kilogram

Description: Average price per kg of the product calculated from observed prices and measured weight (kg) of the transaction units. 1/k Σk (price_per_transaction_unit(k) / transaction_unit_weight_kg(k))

Data type: Numeric

Unit: FCFA/kg

**17. volume_kg**

English name: Volume (kg)

Description: Estimated volume of the produce on the day of the survey. (depense_achat_f_cfa / prix_moyen_kg)

Data type: Numeric

Unit: kg
