# Supplementary material for: Identifying the fresh vegetables foodshed of Brazzaville: A new dataset from a market-based survey
Source: Data Brief. 2026 Jun 20;67:112989. doi: 10.1016/j.dib.2026.112989 (PMC13320381; doi:10.1016/j.dib.2026.112989)
Supplement: Supplementary file 2 [file mmc2.docx]

**PROJET D’APPUI À LA RELANCE DU SECTEUR AGRICOLE (PARSA)**

**Project to Support the Development of the Agricultural Sector**

*QUESTIONNAIRES ENQUÊTE « ORIGINE DES PRODUITS »*

*Questionnaires for the “Product Origin” Survey*

Name of the surveyor:

Date: Time :

Type of market : Wholesale □ Retail □

Name of the market:

Respondent role : Producer□ Collector□ Wholesaler□ Retailer□ Semi-wholesaler□

Respondent gender : Male□ Female□

Selling location type : On a table□ On the floor□ On a store□ Other (describe:) □

| Vegetable type | Production site | Source location | Supplier type | Purchase expenditure (CFA) | Transaction unit |
| --- | --- | --- | --- | --- | --- |
| Amaranth. |  | Market (Name:)  Garden (Name:)  Town (Name:)  Other (Specify:) | Retailer. □  Semi-wholesaler. □  Producer. □  Wholesaler. □  Self-producer □ |  | Bundle  Bed of crop (in the field)  Other (specify:) |
| African nightshade. |  | Market (Name:)  Garden (Name:)  Town (Name:)  Other (Specify:) | Retailer. □  Semi-wholesaler. □  Producer. □  Wholesaler. □  Self-producer □ |  | Bundle  Bed of crop (in the field)  Other (specify:) |
| Bitter nightshade |  | Market (Name:)  Garden (Name:)  Town (Name:)  Other (Specify:) | Retailer. □  Semi-wholesaler. □  Producer. □  Wholesaler. □  Self-producer □ |  | Bundle  Bed of crop (in the field)  Other (specify:) |
| Hibiscus. |  | Market (Name:)  Garden (Name:)  Town (Name:)  Other (Specify:) | Retailer. □  Semi-wholesaler. □  Producer. □  Wholesaler. □  Self-producer □ |  | Bundle  Bed of crop (in the field)  Other (specify:) |
| Hot pepper. |  | Market (Name:)  Garden (Name:)  Town (Name:)  Other (Specify:) | Retailer. □  Semi-wholesaler. □  Producer. □  Wholesaler. □  Self-producer □ |  | Bundle  Bed of crop (in the field)  Other (specify:) |
| Spring onion |  | Market (Name:)  Garden (Name:)  Town (Name:)  Other (Specify:) | Retailer. □  Semi-wholesaler. □  Producer. □  Wholesaler. □  Self-producer □ |  | Bundle  Bed of crop (in the field)  Other (specify:) |
| Lettuce |  | Market (Name:)  Garden (Name:)  Town (Name:)  Other (Specify:) | Retailer. □  Semi-wholesaler. □  Producer. □  Wholesaler. □  Self-producer □ |  | Bundle  Bed of crop (in the field)  Other (specify:) |
| Cabbage |  | Market (Name:)  Garden (Name:)  Town (Name:)  Other (Specify:) | Retailer. □  Semi-wholesaler. □  Producer. □  Wholesaler. □  Self-producer □ |  | Bundle  Bed of crop (in the field)  Other (specify:) |
| Tomato |  | Market (Name:)  Garden (Name:)  Town (Name:)  Other (Specify:) | Retailer. □  Semi-wholesaler. □  Producer. □  Wholesaler. □  Self-producer □ |  | Bundle  Bed of crop (in the field)  Other (specify:) |
| Carrot. |  | Market (Name:)  Garden (Name:)  Town (Name:)  Other (Specify:) | Retailer. □  Semi-wholesaler. □  Producer. □  Wholesaler. □  Self-producer □ |  | Bundle  Bed of crop (in the field)  Other (specify:) |
| Eggplant. |  | Market (Name:)  Garden (Name:)  Town (Name:)  Other (Specify:) | Retailer. □  Semi-wholesaler. □  Producer. □  Wholesaler. □  Self-producer □ |  | Bundle  Bed of crop (in the field)  Other (specify:) |
| Bell pepper. |  | Market (Name:)  Garden (Name:)  Town (Name:)  Other (Specify:) | Retailer. □  Semi-wholesaler. □  Producer. □  Wholesaler. □  Self-producer □ |  | Bundle  Bed of crop (in the field)  Other (specify:) |
| Zucchini |  | Market (Name:)  Garden (Name:)  Town (Name:)  Other (Specify:) | Retailer. □  Semi-wholesaler. □  Producer. □  Wholesaler. □  Self-producer □ |  | Bundle  Bed of crop (in the field)  Other (specify:) |
| Cucumber. |  | Market (Name:)  Garden (Name:)  Town (Name:)  Other (Specify:) | Retailer. □  Semi-wholesaler. □  Producer. □  Wholesaler. □  Self-producer □ |  | Bundle  Bed of crop (in the field)  Other (specify:) |
| Malabar spinach. |  | Market (Name:)  Garden (Name:)  Town (Name:)  Other (Specify:) | Retailer. □  Semi-wholesaler. □  Producer. □  Wholesaler. □  Self-producer □ |  | Bundle  Bed of crop (in the field)  Other (specify:) |
| Green bean. |  | Market (Name:)  Garden (Name:)  Town (Name:)  Other (Specify:) | Retailer. □  Semi-wholesaler. □  Producer. □  Wholesaler. □  Self-producer □ |  | Bundle  Bed of crop (in the field)  Other (specify:) |

Name of the surveyor:

Market: Date :

| Vegetable type | Observed price 1 | Observed weight 1 | Observed price 2 | Observed weight 2 | Observed price 3 | Observed weight 3 | Average price per kg |
| --- | --- | --- | --- | --- | --- | --- | --- |
| Amaranth. |  |  |  |  |  |  |  |
| African nightshade. |  |  |  |  |  |  |  |
| Bitter nightshade |  |  |  |  |  |  |  |
| Hibiscus. |  |  |  |  |  |  |  |
| Hot pepper. |  |  |  |  |  |  |  |
| Spring onion |  |  |  |  |  |  |  |
| Lettuce |  |  |  |  |  |  |  |
| Cabbage |  |  |  |  |  |  |  |
| Tomato |  |  |  |  |  |  |  |
| Carrot. |  |  |  |  |  |  |  |
| Eggplant. |  |  |  |  |  |  |  |
| Bell pepper. |  |  |  |  |  |  |  |
| Zucchini |  |  |  |  |  |  |  |
| Cucumber. |  |  |  |  |  |  |  |
| Malabar spinach. |  |  |  |  |  |  |  |
| Green bean. |  |  |  |  |  |  |  |
